# Comparison of Efficacy Between Endoscopic Cyclophotocoagulation and Alternative Surgeries in Refractory Glaucoma: A Meta-analysis

**DOI:** 10.1097/MD.0000000000001651

**Published:** 2015-10-02

**Authors:** Yangfan Yang, Jing Zhong, Zhongjun Dun, Xiao-an Liu, Minbin Yu

**Affiliations:** From the Department of Glaucoma, State Key Laboratory of Ophthalmology, Zhongshan Ophthalmic Center, Sun Yat-sen University (YY, JZ, X-AL, MY); and Guangdong Provincial Institute of Public Health, Guangzhou, P.R. China (ZD).

## Abstract

Refractory glaucoma refers to uncontrolled intraocular pressure (IOP) despite anti-glaucoma medication and surgical treatment, which remains a challenge to be treated. The objective of this study is to evaluate and statistically compare the clinical efficacy between endoscopic cyclophotocoagulation (ECP) and alternative surgical techniques in the treatment of refractory glaucoma in this article, as a meta-analysis.

Data sources are China Biomedical Database (Sinomed, online version), China National Knowledge Infrastructure (CNKI), Cqvip, Wanfang database, and PubMed.

The randomized controlled trial (RCT) and case–control study literatures evaluating the clinical efficacy between ECP and other surgical techniques were searched electronically from public databases. The methodology quality of the retrieved articles was evaluated according to the RCT or case–control study criteria. The success rate of treatment, intraocular pressure (IOP) and visual acuity were statistically compared. RevMan 5.3 software was used for the meta-analysis.

In total, 6 relevant control studies were selected in this study with a total sampling of 429 cases (429 eyes), including 204 eyes in the ECP group and 225 in the non-ECP group. Meta-analysis demonstrated that the clinical efficacy did not significantly differ between 2 groups (*P* > 0.05). Postoperative IOP was dramatically reduced in both groups. However, it was difficult to evaluate the combined influence of ECP and non-ECP therapies upon IOP reduction.

In conclusion, ECP and non-ECP treatment yielded almost equivalent clinical efficacy in treating refractory glaucoma. The IOP-lowering degree, safety, and incidence of complications remain to be further elucidated by RCTs with a larger sample size.

## INTRODUCTION

Refractory glaucoma refers to uncontrolled intraocular pressure (IOP) despite antiglaucoma medication and surgical treatment. In clinical practice, it remains a challenge to treat refractory glaucoma, such as neovascular glaucoma, aphakic or intraocular lens glaucoma, filtering surgery failure-induced glaucoma, congenital juvenile glaucoma, glaucoma complicated with uveitis, traumatic glaucoma and glaucoma after vitretomy, etc. Conventional filtering surgery and cyclodestruction have been proven to be efficacious in treating glaucoma, whereas yield surgical complications. Although cyclophotocoagulation achieves high clinical efficacy, it has to penetrate into the sclera and destroy the ciliary body under limited surgical field, which inevitably causes injuries to the tissues surrounding ciliary body. Besides, it fails to qualitatively evaluate the severity of ciliary body damage and it tends to yield multiple postoperative complications. Endoscopic cyclophotocoagulation (ECP) is an endoscope-assisted technique to utilize laser energy to destroy partial ciliary body and reduce the production of aqueous humor, thereby lowering the IOP. ECP is performed almost under direct vision with higher accuracy and unique superiority compared with alternative techniques.^1,2^ Recently, multiple studies related to cyclophotocoagulation have been reported. However, few studies have been conducted to evaluate the methodology quality of these investigations. In this study, we evaluated and analyzed the quality of control studies which reported before March 2015.

## MATERIALS AND METHODS

### General Data

Control studies reporting ECP and alternative surgeries in treating refractory glaucoma were retrieved from biomedical bibliographic databases. The cases of neovascular glaucoma, IOL or aphakic glaucoma, filtering surgical failure-induced glaucoma, congenital or juvenile glaucoma, and secondary glaucoma (such as uveitis glaucoma, traumatic glaucoma, secondary glaucoma postcorneal transplantation, etc.) were included and randomly assigned into different groups.

## METHODS

### Publication Search

China Biomedical Database (Sinomed, online version), China National Knowledge Infrastructure (CNKI), Cqvip, Wanfang database, and PubMed were searched for relevant papers published from January 2004 until March 2015 in Chinese or English. Search terms included “endoscopic cyclophotocoagulation,” “transcleral cyclophotocoagulation,” and “glaucoma” in both Chinese and English, tailored to the particular database to guarantee the systematic searching.

### Inclusion Criteria

RCTs or case–control studies reporting ECP and other surgical treatment of refractory glaucoma in Chinese or English without publication category limitations, Clinical trials without gender or race restrictions, and Symptom alleviation as an index for clinical outcome measurement.

### Exclusion Criteria

Noncontrol design, Duplicate publication, Animal studies, Publications not in Chinese or English, Patients in the control group did not undergo surgery, Review, Insufficient raw data, and Descriptive studies without control subjects Lack of demographic data and therapeutic measures of the subjects.

### Clinical Outcome Indexes

At least one of the indexes below was included for observation: IOP-lowering level; category, dosage, and administration approach of IOP-lowering medication; type and incidence of postoperative complications; postoperative recovery of visual acuity; whether visual field defects aggravate the progression of glaucoma.

### Statistical Analysis

RevMan 5.3 software from Cochrance Collaboration was utilized for the meta-analysis. According to the homogeneity test outcomes, *P* < 0.05 was considered as a level of statistical significance, indicating the heterogeneity among different studies.

## RESULTS

### Publication Characteristics

**FIGURE 1:**
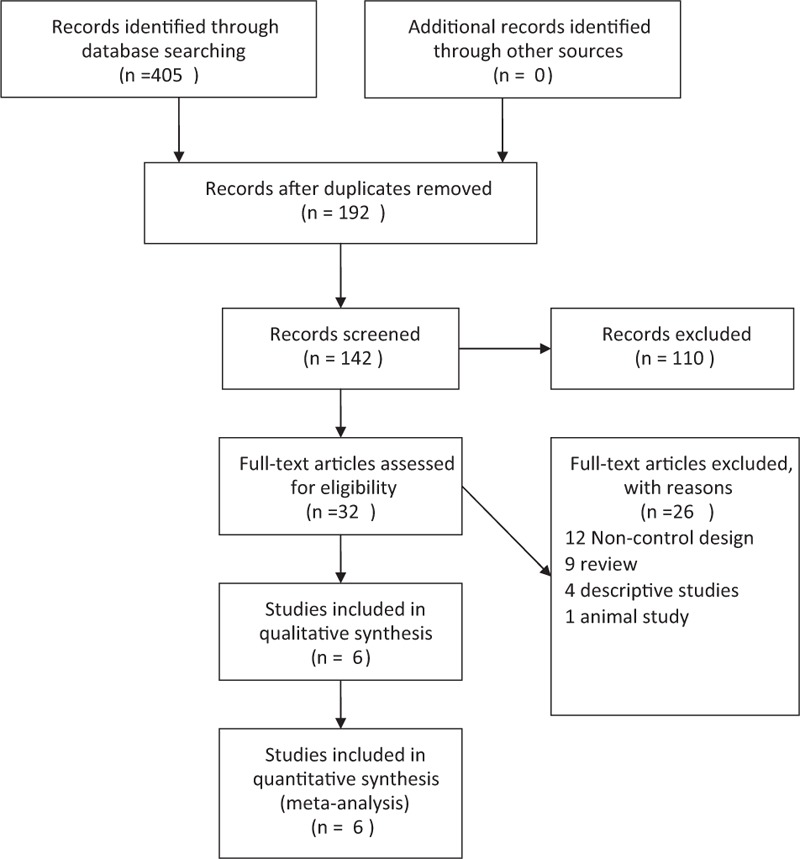
The flow chart of included studies in the meta-analysis.

The flow diagram of study selection procedure is shown in Figure 1. A total of 548 articles were originally identified, including 71 from China Biomedical Database (Sinomed, online version), 13 from China National Knowledge Infrastructure (CNKI), 150 from Cqvip, 126 from Wanfang database, and 45 from PubMed. No additional finding through screening article reference was retrieved. After evaluating articles according to selection, 6 eligible papers (3 in Chinese and 3 in English) which met the inclusion criteria mentioned above were included and 26 were excluded for subsequent analysis, as illustrated in Table 1. Three studies were related to Ahmed shunt implantation, 2 transcleral cyclophotocoagulation, 1 trabeculectomy, and 1 cryotherapy.

**TABLE 1:**
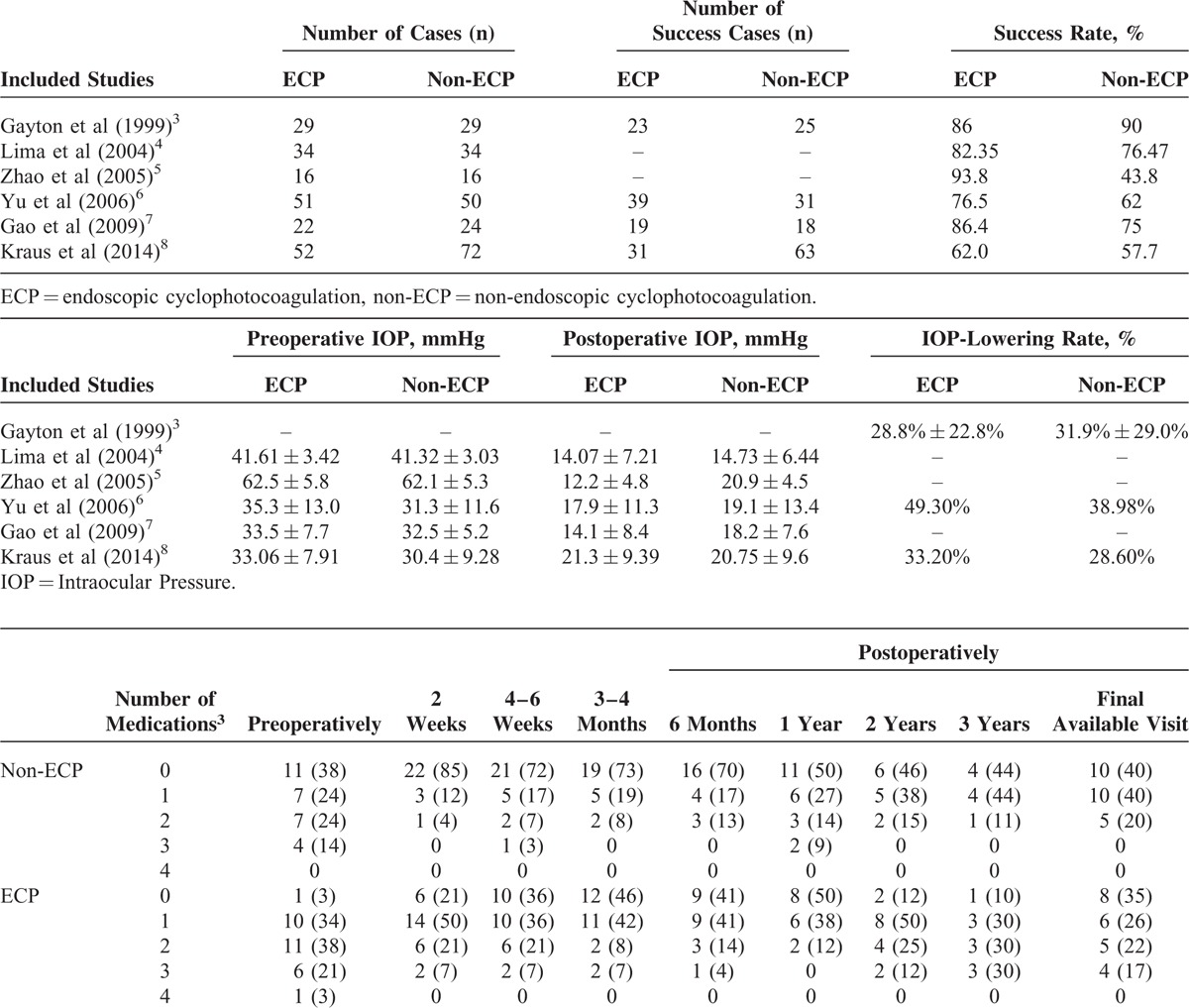
Fundamental Data and Characteristics of Included Publications

### Evaluation of Methodology Quality of Included Publications

Publication bias was not assessed due to the relatively limited quantity of included studies. The type of glaucoma, patients’ age, gender, and mean preoperative IOP between the study and control groups were illustrated in Table 2.

**TABLE 2:**
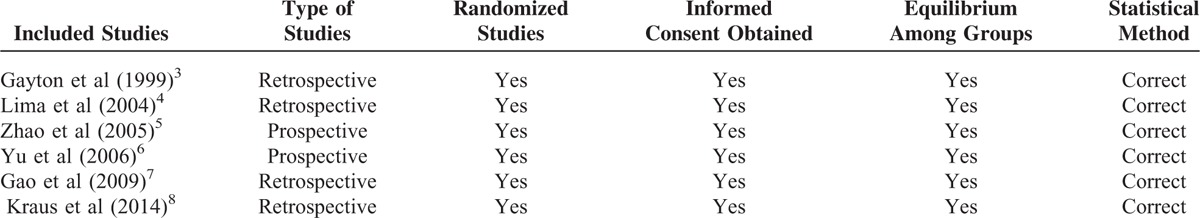
Evaluation of Methodology Quality of Included Publications

### Postoperative IOP-Lowering Level and Antiglaucomatous Medication

All 6 included studies reported the postoperative IOP and IOP-lowering level with standard deviation after ECP and non-ECP in treating refractory glaucoma (429 eyes, 204 in the ECP group and 225 in the non-ECP group). No heterogeneity was noted across different studies. Five publications statistically compared the IOP before and after surgery and merely 1 study obtained the IOP-lowering data. Hence, it was impossible to conduct integrated analysis. Moreover, Gayton et al have listed the amount of glaucoma medications taken by ECP and non-ECP groups. Although more medication was taken by the ECP group at every time period except at 1 year, the difference in mean amount of glaucoma medication was statistically only at 2 weeks postoperatively while insignificantly in other timepoints.^3^

### Success Rate

**FIGURE 2:**
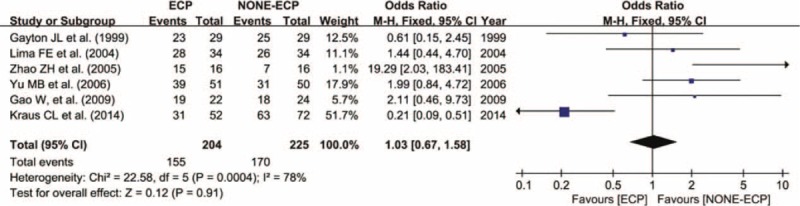
Forest plot and meta-analysis of the occurrence rate of complications. The occurrence rate was similar in ECP and non-ECP (WMD = 1.03, 95% CI: 0.67–1.58, *P* = 0.91). CI = confidence interval, ECP = endoscopic cyclophotocoagulation, M-H = Mantel–Haenszel method, non-ECP = non-endoscopic cyclophotocoagulation (alternative surgeries).

**FIGURE 3:**
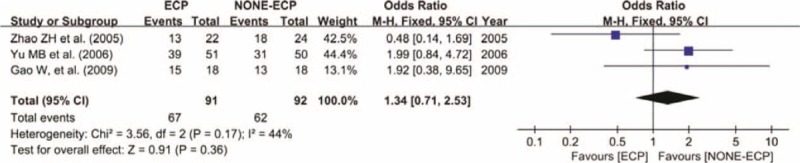
Forest plot and meta-analysis of the occurrence rate of complications. The success rate was similar in ECP and NONE-ECP (WMD = 1.34, 95% CI: 0.71–2.53, *P* = 0.36). CI = confidence interval, ECP = endoscopic cyclophotocoagulation, M-H = Mantel–Haenszel method, non-ECP = non-endoscopic cyclophotocoagulation (alternative surgeries).

All included studies reported the success rate of ECP and non-ECP in treating refractory glaucoma. In total, 429 eyes were included, 204 in the ECP group and 155 successfully treated with a success rate of 75.98%. Among 225 eyes in the non-ECP group, 170 were cured with a success rate of 75.56%. Meta-analysis revealed no statistical significance between 2 groups in terms of success rate. (WMD = 1.03, 95% CI: 0.67–1.58, *P* = 0.91) (Figure 2). The occurrence rate of complications did not significantly differ between ECP and non-ECP groups in treating refractory glaucoma. (WMD = 1.34, 95% CI: 0.71–2.53, *P* = 0.36) (Figure 3).

## DISCUSSION

### Progress of Refractory Glaucoma Treatment

Refractory glaucoma, including neovascular glaucoma, aphakic or intraocular lens glaucoma, filtering surgery failure-induced glaucoma, congenital juvenile glaucoma, glaucoma complicated with uveitis, traumatic glaucoma and glaucoma after vitretomy, etc., still remains a challenge to be treated. At present, the treatment methods below are mainly employed to treat refractory glaucoma: filtering surgery combined with antimetabolites;^1^ aqueous shunt implantation including Molteno and Baerceldt drainage shunt without a valve and Krupin, Ahmed and OptiMed valve implantation, etc.^2^ suprachoroidal drainage (filtering surgery);^9^ Vitreoretinal surgery is applied to treat malignant glaucoma and neovascular glaucoma induced by retinal ischemia change, etc.;^10^ intraocular injection of anti-VEGF agents is utilized for the management of neovascular glaucoma and glaucoma accompanied with uveitis;^11^ Cyclodestruction consists of scleral diathermy, cyclocryotherapy and cyclophotocoagulation, etc. Scleral diathermy and cyclocryotherapy tend to result in postoperative atrophy of eyeball due to severe pain during and after surgery, which are rarely applied in clinical practice.^12^ Currently, cyclophotocoagulation and ECP are commonly adopted to treat refractory glaucoma.^13^

### Application of Endoscopic Cyclophotocoagulation in the Management of Refractory Glaucoma

Although conventional filtering surgery or cyclodestruction has been proven to be efficacious in certain cases of refractory glaucoma, these therapies are likely to cause multiple surgical complications and yield poor clinical efficacy.^3,4^ ECP functions by direct photocoagulation of ciliary process assisted by eye endoscope. During ECP procedures, the pigment epithelium generates thermal effect after absorption of laser energy, destroys ciliary epithelium, and decreases the secretion of aqueous humor, thereby lowering the IOP. ECP is able to accurately locate the anatomical site, conduct quantitative photocoagulation of ciliary body, avert the influence of refractive media opacity, cause mild injuries to surrounding tissues and exert significant effect upon IOP lowering, etc.^13^ Hence, ECP has been recognized as a widespread treatment of a variety of refractory glaucoma, such as IOL and aphakic refractory glaucoma. It could be performed combined with alternative surgeries, such as phacoemulsification and vitrectomy, etc.^14^ Lima et al^15^ recommended ECP as the primary therapy of glaucoma, and ECP combined with phacoemulsification for coexisting cataract and glaucoma.

Relevant previous studies reported that the success rate of ECP ranged from 70% to 90%.^4,16,17^ Yu et al^18^ have conducted a series of investigations analyzing the surgical approach, technical parameter, surgical indications, clinical efficacy, and surgical safety since the year of 2003.^19,20^ They reported that postoperative IOP was lowered by 49.3% after ECP and the success rate at postoperative 12 months achieved to 66.67%.^18^ The IOP-lowering level after ECP was significantly higher compared with that transcleral cyclophotocoagulation and the 1-year success rate of ECP was 66.7%, significantly higher than 48.0% of transcleral cyclophotocoagulation.^19^ Surgical complications included postoperative uveitis reaction, ocular hypertension, and hyphema. Most of surgical complications occurred early after surgery and could be alleviated and cured by active and effective treatment.^20^

### Advantages and Limitations of This Meta-Analysis

In recent years, endoscope has rapidly emerged as a tool in the field of ophthalmology. In addition, ECP has been proven to serve as a safe and effective surgical option with high reproducibility, precise localization, mild trauma, and few complications. Moreover, it could be performed in combination with alternative intraocular surgeries. A substantial quantity of studies has been conducted to describe and observe the clinical outcomes of ECP, whereas treatment parameters and postoperative outcomes remain significantly diverse. Thus, this meta-analysis was designed to evaluate the clinical value of ECP in the management of refractory glaucoma, add evidence-based medicine data to clinical settings and provide guidance for the selection of clinical treatment for refractory glaucoma. Six RCTs reporting ECP and non-ECP in treating refractory glaucoma were included. A total of 429 eyes, 204 in the ECP and 225 in the non-ECP groups, were assessed. Patients diagnosed with varying types of refractory glaucoma were recruited, such as neovascular glaucoma, traumatic glaucoma, uncontrolled IOP after repeated surgeries, aphakic glaucoma, secondary glaucoma after corneal transplantation, secondary glaucoma following vitrectomy, etc. Non-ECP interventions included transcleral cyclophotocoagulation, trabeculectomy, drainage implantation, and cryotherapy. Meta-analysis revealed that ECP and non-ECP shared similar surgical efficacy with no statistical significance (*P* > 0.05).

The present meta-analysis has some limitations that should be acknowledged. First, merely 6 eligible studies were included and the number of observational parameters was relatively small. Hence, more clinical data and further studies in the future will be necessary to allow a safe generalized treatment of the refractive glaucoma. Second, heterogeneity might arise between the ECP and non-ECP groups when multiple surgical methods were adopted in the non-ECP group, which probably affects the analysis outcomes. Third, the data requirement is inconsistent between observational and analytical studies. In an observational study, only postoperative IOP value should be obtained to assess the IOP-lowering effect and surgical success rate. However, in a meta-analysis, both preoperative and postoperative IOP data are required to calculate the changes in IOP before and after surgery. In certain included studies, preoperative IOP values are lacking, which affects subsequent analysis. Fourth, refractory glaucoma has a variety of primary diseases and complications. ECP is commonly conducted in combination with alternative surgeries, such as phacoemulsification and vitrectomy, to enhance the surgical efficacy and relieve pain. However, those studies were not included in this meta-analysis. Hence, the test potency in this study is limited and the conclusion remains to be further validated. Previous studies indicated that long-term use of medication is required to effectively lower the IOP in certain patients.^21^ In this meta-analysis, preoperative and postoperative medical management has been reported only in 2 studies, which demonstrated that the dosages of drugs were reduced after surgery while ECP has not shown obvious superiority compared with other surgeries. Moreover, the machine's cost is considered to be a significant limitation which restricts the development of surgical technique. Last but not least, it is possible to conclude the presumed effective advantages in the future such as a better postoperative control of the IOP lowering, when much more data and significant results could be achieved to demonstrate them.

Taken together, ECP is a novel safe and efficacious treatment of refractory glaucoma. It is a challenging task to conduct large-scale randomized case–control clinical studies considering the complex pathogenesis and primary diseases of refractory glaucoma and combined application of ECP with other surgical approaches. Therefore, clinical observational studies with rational design and complete record should be included to evaluate the clinical application of ECP in the management of refractory glaucoma. Long-term visual function, quality of life, and cost-benefit ratio should be greatly emphasized.
